# Neutralizing tumor-related inflammation and reprogramming of cancer-associated fibroblasts by Curcumin in breast cancer therapy

**DOI:** 10.1038/s41598-023-48073-w

**Published:** 2023-11-26

**Authors:** Elnaz Jalilian, Firoozeh Abolhasani-Zadeh, Ali Afgar, Arash Samoudi, Hamid Zeinalynezhad, Ladan Langroudi

**Affiliations:** 1 Department of Medical Immunology, School of Medicine, Kerman University of Medical Sciences, Pajoohesh Sq, Kerman, Iran; 2 Department of Surgery, School of Medicine, Kerman University of Medical Sciences, Kerman, Iran; 3 Departmeny of Parasitology and Mycology, School of Medicine, Kerman University of Medical Sciences, Kerman, Iran

**Keywords:** Breast cancer, Cancer microenvironment, Inflammation, Tumour immunology

## Abstract

Tumor-associated inflammation plays a vital role in cancer progression. Among the various stromal cells, cancer-associated fibroblasts are promising targets for cancer therapy. Several reports have indicated potent anti-inflammatory effects attributed to Curcumin. This study aimed to investigate whether inhibiting the inflammatory function of cancer-associated fibroblasts (CAFs) with Curcumin can restore anticancer immune responses. CAFs were isolated from breast cancer tissues, treated with Curcumin, and co-cultured with patients' PBMCs to evaluate gene expression and cytokine production alterations. Blood and breast tumor tissue samples were obtained from 12 breast cancer patients with stage II/III invasive ductal carcinoma. Fibroblast Activation Protein (FAP) + CAFs were extracted from tumor tissue, treated with 10 μM Curcumin, and co-cultured with corresponding PBMCs. The expression of smooth muscle actin-alpha (α-SMA), Cyclooxygenase-2(COX-2), production of PGE2, and immune cell cytokines were evaluated using Real-Time PCR and ELISA, respectively. Analyzes showed that treatment with Curcumin decreased the expression of genes α-SMA and COX-2 and the production of PGE2 in CAFs. In PBMCs co-cultured with Curcumin-treated CAFs, the expression of FoxP3 decreased along with the production of TGF-β, IL-10, and IL-4. An increase in IFN-γ production was observed that followed by increased T-bet expression. According to our results, Curcumin could reprogram the pro-tumor phenotype of CAFs and increase the anti-tumor phenotype in PBMCs. Thus, CAFs, as a component of the tumor microenvironment, are a suitable target for combination immunotherapies of breast cancer.

## Introduction

Chronic inflammation and tumor-related inflammation are known as one of the hallmarks of cancer involved in the development and progression of cancer^1,2^. Clinical and experimental studies demonstrate that activating inflammatory pathways leads to destructive inflammation in cancer cells and the tumor microenvironment (TME), resulting in phenotypic and functional changes contributing to cancer promotion^3^. In this regard, a few therapeutic candidates, including medicinal herbs with anti-inflammatory properties, have been analyzed for their potential applications^4^.

Chronic inflammation is regulated by specific signaling pathways acting as suppressors or activators^5^. In the TME, various inflammatory stimuli prompt the activation of transcription factors such as NF-κB, STAT3, and HIF-1α and are, in turn, responsible for the production of cytokines, growth factors, and activation of immune and stromal cells^6^. Activated by oxidative stress and pro-inflammatory cytokines, NF-κB primes inflammation-related cellular transformation via the expression of various genes including anti-apoptotic proteins (BCL-XL, BCL-2)^7^, cytokines (TNF-α, IL-1β, IL-6, IL-8)^8^, inflammatory enzymes (iNOS, and COX-2), active molecules in invasion and metastasis, such as adhesion molecules and matrix metalloproteases (MMPs)^9^, cell cycle molecules (c-MYC and cyclin D1)^10^, and angiogenic factors (VEGF and angiopoietin)^11^. Therefore, activation of inflammation participates in all stages of cancer progression.

Among the inflammation-related expressed genes, Cyclooxygenases (COXs) are a family of myeloperoxidases; with prostaglandins as their main products, they regulate physiological functions such as vasodilation, renal homeostasis, fever, pain sensitivity, and inflammation. The induced isoform Cyclooxygenase-2 (COX-2), expressed in damaged cells, placenta, and during inflammation, is over-expressed in many cancer cells such as the lung, prostate, breast, and stromal cells of TME^12^. Various studies suggest that COX-2 is involved in tumor survival, growth, invasion, and metastasis. COX-2 is also involved in the immunosuppression observed in the tumor microenvironment^13^. It has been shown that in pancreatic and lung cancer, increased levels of prostaglandin E2 (PGE2) augment the function and number of regulatory T cells (Treg)^14^. CD4^+^, CD25^+^, and FoxP3^+^ Tregs play an essential role in tumor immune tolerance and failure of immunotherapy by secreting immunosuppressive cytokines such as TGF-β and IL-10^15^. It has been observed that COX-2 expression increases when tumor cells are cultured with fibroblasts, suggesting a reciprocal increase. In the presence of immune factors of the TME, specifically TGF-β and IL-1β, fibroblasts transform into an active form called Cancer-associated Fibroblasts (CAFs)^16^. CAFs increase the expression of inflammation-related genes and are responsible for immunosuppression in the TME^17^. The phenotypic changes in CAFs are diverse and various subtypes expressing different molecules are described which among them include increased expression of α-smooth muscle actin (α-SMA), Fibroblast activation protein (FAP), platelet-derived growth factor receptors α and β (PDGFRα and β), podoplanin (PDPN), caveolin-1 (CAV1), and fibroblast-specific protein 1 (FSP1)^18–20^. However, a specific marker is yet to be characterized for identifying CAFs, and for now, a selection of positive and negative markers are utilized. On the other hand, studies acknowledge the immunosuppressive activity of different subtypes^21^. Studies have shown that CAFs increase the number and function of Tregs through TGF-β cross-talk. Also, CAFs are responsible for the decreased function of CD8 + T cells and natural killer (NK) cells in the tumor microenvironment^16^

Several studies suggest combining chemotherapy agents with Anti-inflammatory treatments has beneficial effects on treatment responses and prognosis (For review, refer to^4^). Several anti-inflammatory agents, including NSAIDs^22^, Corticosteroids^23^, COX-2 Inhibitors^24^, and Natural Products such as Curcumin, Ginseng, and Garlic^25,26^ have been studied in combination with chemotherapies. Among the traditional medicinal compounds, Curcumin (diferuloylmethane) is a non-toxic polyphenol derived from the plant Curcuma longa with therapeutic properties such as antioxidant, analgesic, anti-inflammatory, antiseptic, and anticancer activities^27^. Curcumin functions via negative regulation of NF-κB, affecting a variety of signaling pathways and molecules such as COX-2^28^, cyclin D1^29^, VEGFR, and / mTOR phosphoinositol-3 (PI) 3 / Akt^30^.

Based on the critical function of COX-2 and PGE2 in inflammation-related carcinogenesis, inhibition of PGE2 by Curcumin in CAFs provides a basis for anticancer and anti-inflammatory applications. In this study, we aimed at the properties of Curcumin in reducing CAF phenotype and its effects on the TH1 vs. Treg phenotype of PBMCs co-cultured with Curcumin-treated CAFs.

## Material and methods

### Patients and sample collection

Twenty-nine breast cancer tumor samples were collected from Bahonar Hospital, Kerman, Iran, between September 2019 and August 2020. The inclusion criteria were new case breast cancer in stages II or III of invasive ductal carcinoma (IDC) with no prior treatment with chemotherapy or radiotherapy and no history of autoimmune disorders. Breast cancer tumor samples were collected during surgery and maintained in a transfer media containing culture media, penicillin (100 units/mL), streptomycin (100 mg/mL), and Amphotericin B (2.5 ng/mL) during transfer to the Immunology cell culture lab at Kerman medical university. Informed consent was obtained from all patients, and they received standard care and treatment.

Simultaneously, 10 ml of blood was collected from each patient and transferred on ice to the Immunology cell culture lab at Kerman Medical University. The samples were processed immediately, and peripheral blood mononuclear cells (PBMCs) were isolated using the Ficol (Biosera) separation technique. PBMCs were counted, and viability was evaluated using trypan blue staining. Isolated PBMCs were frozen in liquid nitrogen until further analysis.

### CAF isolation and characterization

Breast cancer tumor samples were collected in transfer media and processed immediately. Tumor samples were washed in cold PBS, and excess tissues such as fat, necrotic tissue, and visible vasculature were removed. The tissue was minced into 1–3 mm pieces and placed into a T75 tissue culture flask (SPL, South Korea) containing DMEM (Gibco, Germany) supplemented with 30% FBS (Gibco, Germany). The outgrowth of fibroblastic colonies started after two days, and after 3 Weeks, the flask had reached 80% confluence.

Isolated CAFs were immune-stained for the expression of FAP. Briefly, CAFs were cultured in 4-well tissue culture plates (SPL, South Korea) and fixed with 4% paraformaldehyde (PFA) (Sigma Aldrich, MO). Cells were washed with 0.1% PBS-tween 20, blocked with 5% goat serum, and stained with rabbit anti-human FAP antibodies (eBioscience) overnight at 4ºC. Afterward, cells were washed with 0.1% PBS-tween 20, stained with FITC conjugated goat anti-rabbit IgG secondary antibody (eBioscience), and counterstained with DAPI (Sigma Aldrich, MO). Cells were visualized using a fluorescent microscope (Carl Zeiss Microscopy GmbH, Jena, Germany).

### Cell viability and chemosensitivity assay

3(4,5-dimethylthiazol2-yl)-2,5-diphenyltetrazolium bromide (MTT; Melford, UK) assay was used to assess cellular response to Curcumin. Previous studies have shown that CAFs are sensitive to high doses of Curcumin (over 20uM) and time over 48 h^31–33^. As this study aimed to evaluate the inhibitory effects of Curcumin on COX-2 in CAFs, the treatment was not extended over 24 h to avoid further damage to cell viability. Three isolated CAF cells were seeded (8*10^3^ cells per well) in 96-well plates (SPL, South Korea) and exposed to varying concentrations (0, 5, 10, 20 and 25 μM) of Curcumin. After 24h, the optical density was determined at 570 nm using an ELISA plate reader (Biotek 800, Vermont, USA). The cellular viability was calculated with the following formula: % viability = (mean OD of treated cells *100)/ (mean OD of control cells). Each assay was performed in triplicate in three independent experiments, and the 50% inhibitory concentration (IC50) was calculated using sigmoidal log(concentration)-inhibition curve fitting models using GraphPad 9.0 software (GraphPad, CA).

### Treatment with CUR and co-cultures

Curcumin (Sigma, USA) powder was used to prepare a stock solution of 100 µM in DMSO and stored at -20°C until use. Further dilution of the stock solution was made in PBS to keep the final concentration of DMSO below 0.5% in cell culture. Isolated CAFs were cultured in 24-well plates at 10^5^ cells/well and treated with 10µM of Curcumin (CUR-CAF), the equivalent concentration of DMSO, or PBS for 24 h, which after the cells were washed with media, and Curcumin was removed and CAFs were inactivated with Mitomycin C. These cells were used in co-coculture experiments for proliferation, cytokine production, and gene expression assays.

### Evaluation of PBMC proliferation and cytokine production

The ability of Curcumin-treated CAFs to affect immune cell proliferation, as the initial response of immune cell activation, was evaluated using MTT [3-(4,5-dimethylthiazol-2-yl)-2,5-diphenyltetrazolium bromide] assay (Melford, UK). PBMCs were treated for 4 h with 5µg/mL Phytohemagglutinin (PHA) (GIBCO, Germany), washed and were cocultured with CAFs in cell–cell contact at three ratios of 1/5, 1/10, and 1/50 (CAFs/ PBMCs) in RPMI (Gibco, Germany) supplemented with 10% FBS_._ After 72 h, PBMC proliferation was assessed. Briefly, MTT was added at 5mg/ml concentration, and after 4 h of incubation, the media was replaced with DMSO. The formazan crystal formation was evaluated using a plate reader at 570 nm. Co-culture ODs were corrected with mono-CAF culture. PBMCs were activated by 5µg/mL PHA as a positive control, and the negative control was untreated PBMCs. The optical density was recorded, and PBMC proliferation was calculated and expressed as a stimulation index (SI) as follows:

$$StimulationIndex = \left( {OD_{cc - P} - OD_{CAF} } \right)/\left( {OD_{PBMC - P} - OD_{PBMC} } \right)$$

where OD_cc-P_ is the optical density of co-culture of CAFs and stimulated PBMCs, OD_CAF_ is the optical density of CAFs alone, OD_PBMC-P_ is the optical density of stimulated PBMCs alone, and OD_PBMC_ is the optical density of unstimulated PBMCs.

Curcumin-treated CAFs were co-cultured with PBMCs at a 1/10 (CAFs/ PBMCs) ratio in RPMI supplemented with 10% FBS for cytokine evaluation. After 48 h, the supernatant was collected, and the levels of IFN-ɣ, IL-4, IL-10, TGF-β1, and PGE2 were assessed by ELISA (DuoSet ELISA Development kit, R&D Systems, Minneapolis, MN, USA). All procedures were followed according to the manufacturer’s protocol.

### qRT-PCR and gene expression

Gene expression analysis was performed on CAFs and PBMCs before and in treatment with Curcumin. Total cellular RNA was extracted using the Trizol technique (Yekta Tajhiz Azma, Tehran, Iran). Random hexamer-primed reverse transcription (Yekta Tajhiz Azma Tehran, Iran) was performed on aliquots (1 µg) of total RNA. The resulting cDNA was used for Real-Time PCR amplification. Primers (Metabion, GmbH) for PTGS2 prostaglandin-endoperoxide synthase 2 (COX-2), alpha-smooth muscle actin (α-SMA), and beta-actin in CAFs and Glyceraldehyde 3-phosphate dehydrogenase (GAPDH), T-box protein expressed in T cells (T-bet), Forkhead box-p3 (FoxP3), and GATA Binding Protein 3 (GATA-3) for PBMCs were synthesized based on the reported sequences and are as follows:

- Beta-actin: Forward**5ʹ-**CTT CCT TCC TGG GCA TG-**3ʹ,** Reverse**5ʹ-**GTC TTT GCG GAT GTC CAC**-3ʹ;**
- Alpha smooth muscles actin: Forward**5ʹ-**GGACGCACAACTGGC-**3ʹ,** Reverse**5ʹ-**CGGACAATCTCACGCT**-3ʹ;**
- COX-2 (*ptgs2*): Forward**5ʹ-**CGGACAGGATTCTATGGA-**3ʹ,** Reverse**5ʹ-**TCTGGATGTCAACACATAACT**-3ʹ;**
- GAPDH: Forward**5ʹ-**CTCTCTGCTCCTCCTGTTCG-**3ʹ,** Reverse**5ʹ-**ACGACCAAATCCGTTGACTC**-3ʹ;**
- T-bet: Forward**5ʹ-**TGCTCCAGTCCCTCCATAAGT A-**3ʹ,** Reverse**5ʹ-**TCTGGCTCTCCGTCGTTC A**-3ʹ;**
- GATA-3: Forward**5ʹ-**GAGACAGAGCGAGCAAC-**3ʹ,** Reverse**5ʹ-**CTCGGGTCACCTGGGTA**-3ʹ;**
- FOXP3: Forward**5ʹ-**TGGCATCATCCGACAAG-**3ʹ,** Reverse**5ʹ-**AGGAACTCTGGGAATGTG**-3ʹ.**

Real-time PCR reaction mixtures (final volume of 30 µl) contained the following: 1 µl cDNA, 50 pmol of each primer, 3 µl of 200 µM dNTP, and 1U Taq-DNA polymerase (MBI Fermentas Inc., Burlington, ON). Amplification conditions were as follows: 40 cycles of 95°C for 30 s, 60 °C for 30 s, and 72 °C for 30 s, followed by 72 °C incubation for 10 min (Corbett Life Sciences, Australia). The results of primer amplification were analyzed using Rotor-Gene 6000® (Qiagen AG Hilden, Series Software 1.7, build 34) and REST software version 2009^34^.

### Statistical analysis

All data are presented as mean ± SD otherwise stated. After performing the Shapiro–Wilk test for normality, data were analyzed by ANOVA-Tukey post hoc test or Kuskal-Wallis test using SPSS v.19 software. GraphPad Prism v.8 was used for visualization. For paired comparisons, the paired T-test was used. The relative expressions were analyzed using REST software v. 2009. A P-value less than 0.05 was considered statistically significant. All experiments detailed above were performed under relevant guidelines and regulations of the Kerman University of Medical Sciences.

### Ethical statement

This study was approved by the ethical committee of the Kerman University of Medical Sciences with the reference number IR.KMU.REC.1398.326. Informed consent was obtained from all participants before the procedure.

## Results

### Patients and PBMC and CAF isolation

**Figure 1:**
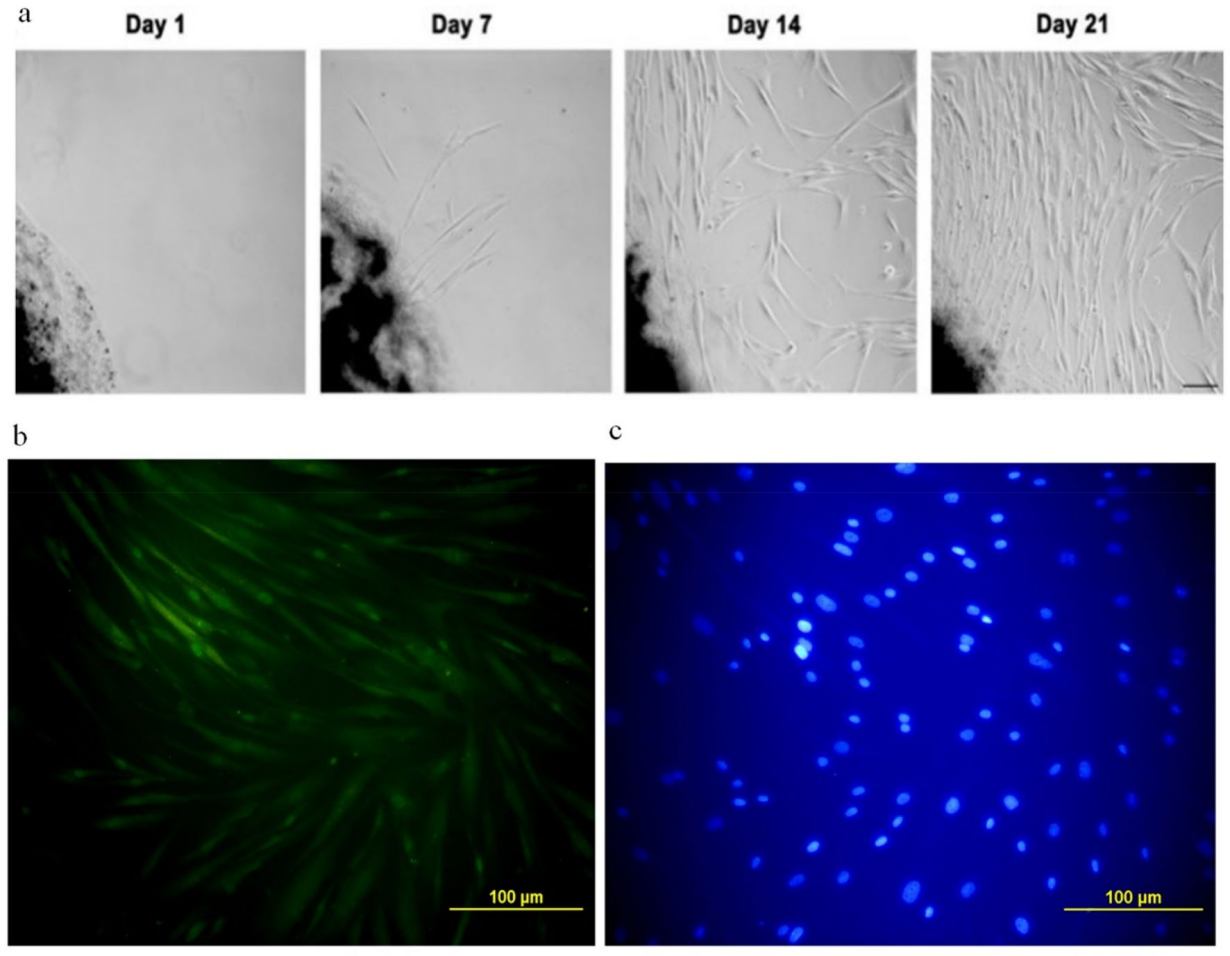
Isolation and characterization of CAFs. (**a**) The outgrowth of fibroblastic cells from tumor tissue began on day two and reached 80% confluence on day 21. These cells were passaged and maintained for Curcumin treatment and co-culture experiments. (**b**) Passage 2 or 3 CAFs were used in all experiments. Fibroblastic colonies were cultured in 4-well plates and immune stained with rabbit anti-human FAP, and (**c**) nuclei were counterstained with DAPI. Cells showed expression of FAP.

Twenty-nine breast cancer tissues diagnosed with stage II or III IDC between September 2019 and August 2020 were collected, from which 12 samples yielded tangible CAFs and were included in this study. The patient's demographic data is shown in Table 1. Tumor tissue and blood samples were collected from patients undergoing surgery by a specialized surgeon. The immunostaining results showed that all isolated CAFs were positive for expression of FAP, indicative of CAF phenotype (Fig. 1). The outgrowth of fibroblastic cells from tumor tissue began on day two and reached 80% confluency on day 21. These cells were passaged and maintained for Curcumin treatment and co-culture experiments. Passage 2 or 3 CAFs were used in all experiments. Trypan blue viability assay of PBMCs revealed 99% viability after isolation and 85% after de-freezing, before co-culture. Each patient's CAFs were co-cultured with the respective PBMCs.

**Table 1:** Demographic information of patients and tumor type entered the study.

Variable	Mean ± SD
Age	46.7 ± 9
Max	62
Min	34
Tumor size	*n* (%)
T1	2 (16.6)
T2	10 (83.4)
Lymph node involvement	*n* (%)
N0	7 (58.2)
N1	3 (25)
N2	1 (8.4)
N3	1 (8.4)
TNM staging	*n* (%)
At least IIA	9 (75)
At least IIB	2 (16.6)
At least IIIC	1 (8.4)
Differentiation	*n* (%)
Well	2 (16.6)
Moderate	3 (25)
Moderate/poorly	4 (33.4)
Poorly	3 (25)

The results are based on pathology specialist lab reports.

### Cell viability assay and IC_50_ calculations

**Figure 2:**
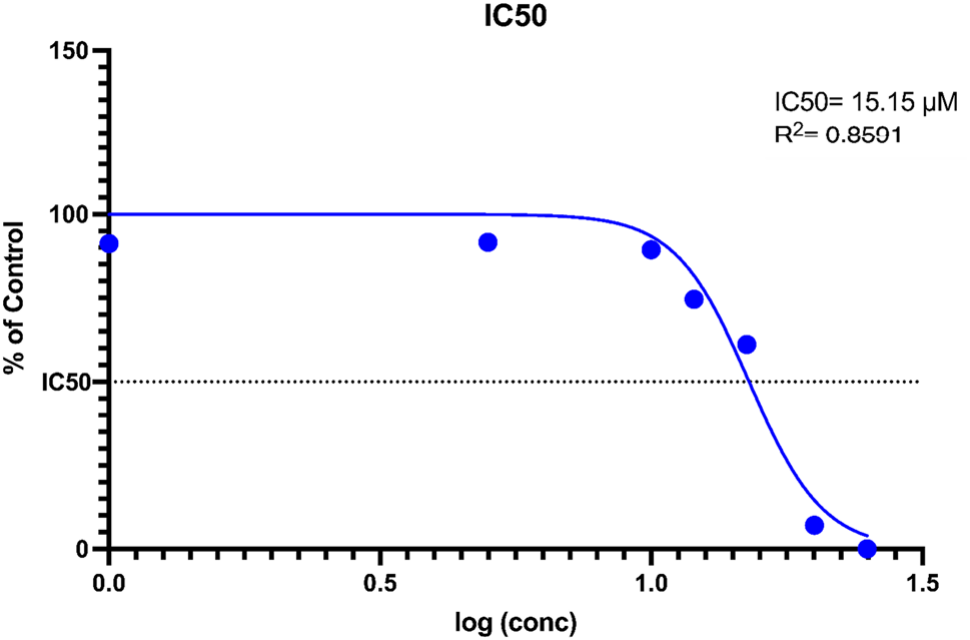
IC_*50*_ value of Curcumin in 3 isolated CAFs. CAFs were cultured in standard conditions and treated with different concentrations of Curcumin. Log(inhibition)-curve fitting model was used to calculate the IC_*50*_ value. Data represents the mean of triplicates of 3 isolated CAFs. Results showed that the IC_*50*_ = 15.15 μM ± 1.15 with an R^2^ of 0.8591.

To determine the appropriate dose of Curcumin, the IC50 value was evaluated in CAFs to assess chemosensitivity. The MTT assay showed that the isolated CAF cells were sensitive to high doses of Curcumin. As the results of the curve-fitting analysis showed, the IC50 of Curcumin was 15.15 μM (95% CI 14.03–16.34) (Fig. 2). In addition, reports indicate that the inhibitory concentration of Curcumin on COX-2 starts at 5 μM and as early as 6 h^35^. Therefore, considering the IC50 value and inhibition of COX-2, the chosen optimum concentration of Curcumin used to treat CAFs was 10 μM for 24 h.

### Treatment with Curcumin was effective in the suppression of CAF phenotype

**Figure 3:**
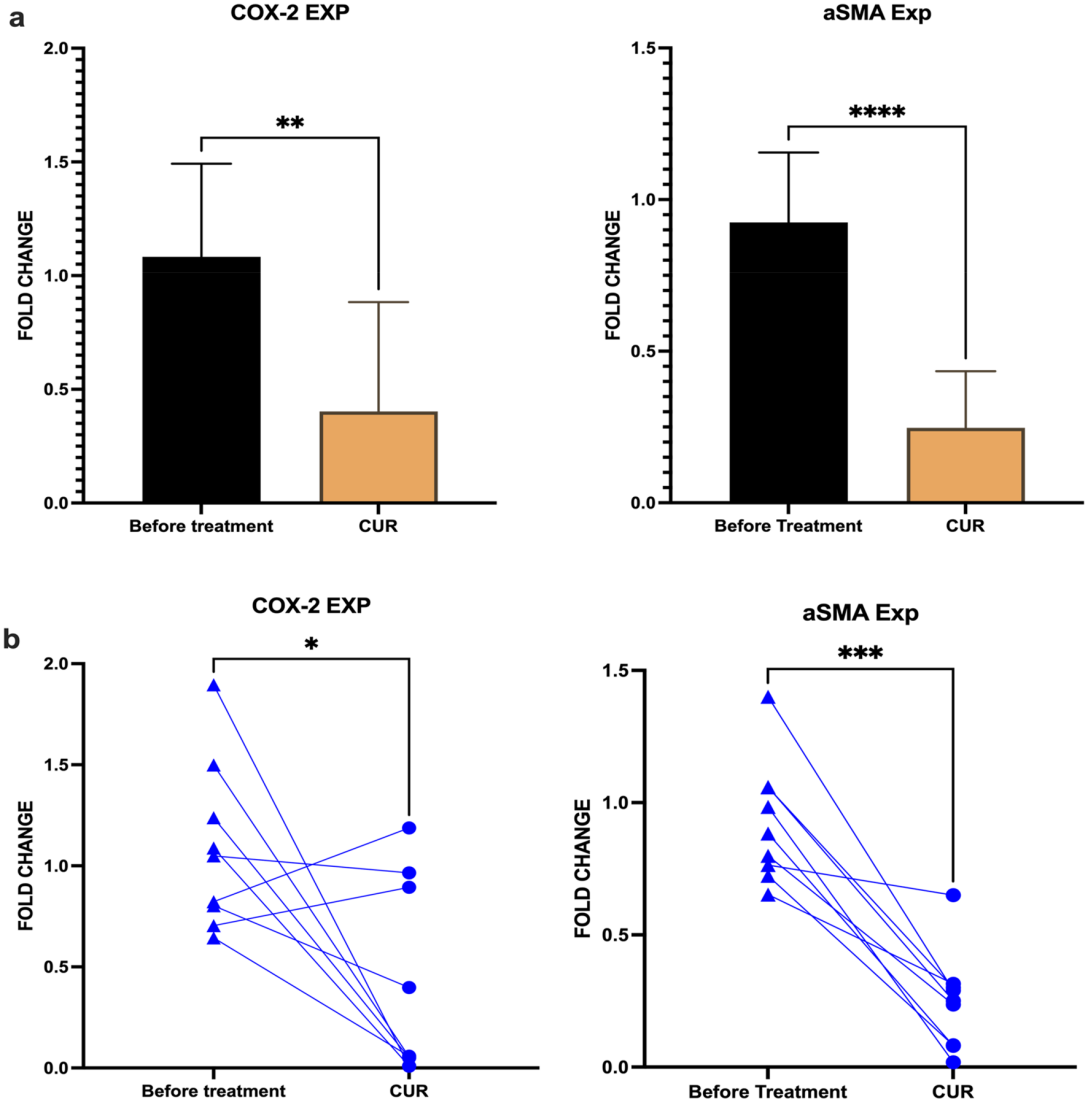
Effect of Curcumin on CAF phenotype. (**a**) The effect of Curcumin treatment on the mRNA expression of COX-2 and αSMA was analyzed using Real-Time PCR. Results showed that CUR could decrease the functional markers associated with CAF phenotype, COX-2 and αSMA. (**b**) Each sample's gene expression fold changes were analyzed using the paired T-test in three replicates. Individual comparisons also showed a significant reduction in the expression of COX-2 and α-SMA. Data represents mean ± SD. Significant changes are indicated with asterisk *P-value < 0.05, **P-value < 0.01, ***P-value < 0.001.

The changes in CAF phenotype and function in treatment with Curcumin were evaluated using cytokine production and gene expression by ELISA and Real-Time PCR, respectively (Fig. 3). Results showed that the production of PGE2 and TGF-β decreased significantly. CAFs were assessed for the expression of CAF phenotype genes, including α-SMA and COX-2, using real-time PCR, which indicated that their mRNA level decreased in treatment with Curcumin.

**Figure 4:**
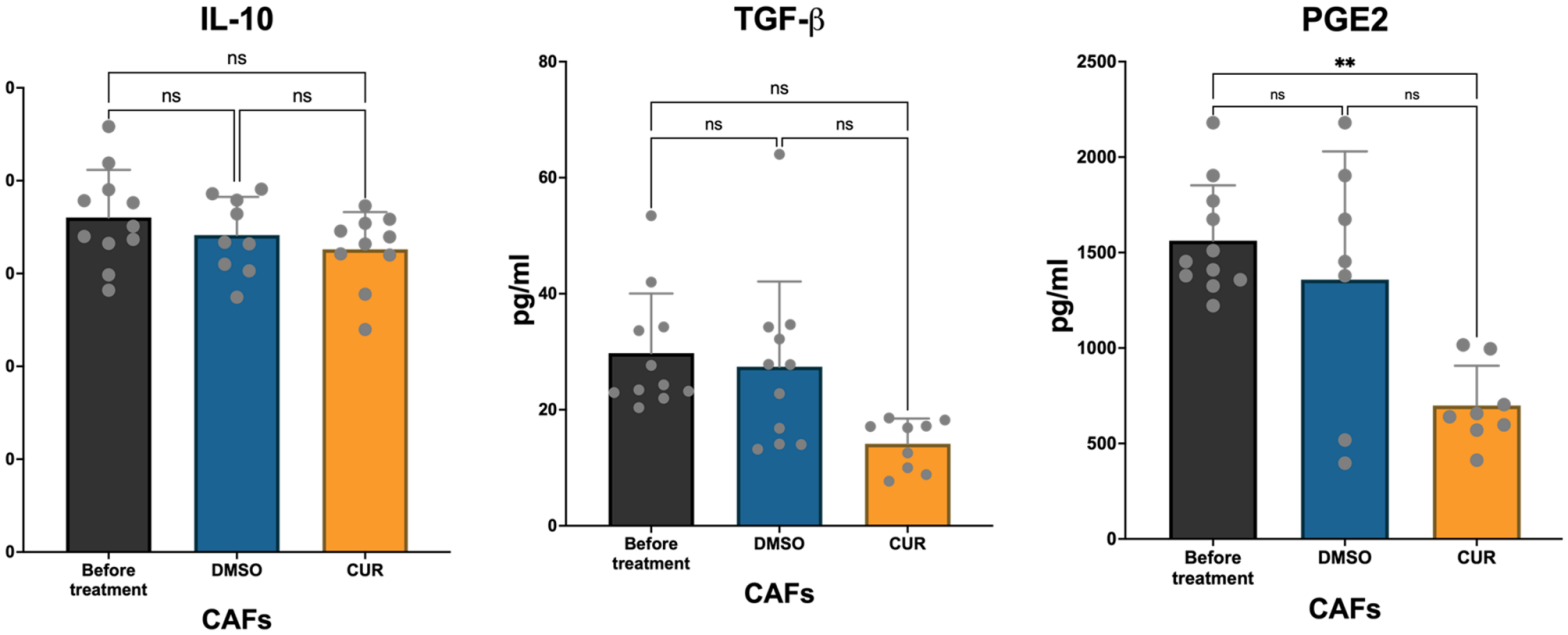
Effect of Curcumin on CAFs' cytokine production. Production of cytokines IL-10, TGF-β, and PGE2 was evaluated using ELISA. Results indicated that after treatment with Curcumin, the production of cytokines associated with CAF phenotype and function, including TGF- β, was significantly reduced. The production of PGE2 from Curcumin-treated CAFs also showed a significant decrease. However, the level of IL-10 was unaffected. Data represents mean ± SD. Significant changes are indicated with asterisk *P-value < 0.05, **P-value < 0.01, ***P-value < 0.001.

Gene expression analysis using real-time PCR indicated that the mRNA level of α-SMA and COX-2 genes decreased in Curcumin-treated CAFs (P-value = 0.017 and 0.03, respectively). Curcumin treatment inhibited the expression of CAF phenotype-related genes by as much as 50%. Whether extended treatment time presented higher inhibition rates requires further studies. PGE2 was evaluated in the supernatant of Curcumin-treated, DMSO-treated, and non-treat CAFs. The decrease in the production of PGE2 in Curcumin-treated CAFs was as much as three-fold (Fig. 4). TGF-β, another cytokine product of CAFs, was also inhibited by Curcumin (P-value for PGE2 = 0.009 and TGF-β = 0.04). Results revealed that treatment with Curcumin did not significantly affect the production of IL-10 from CAFs (P value > 0.05).

### Treatment with Curcumin was able to restore proliferation in PBMC and increase the TH1-phenotype

**Figure 5:**
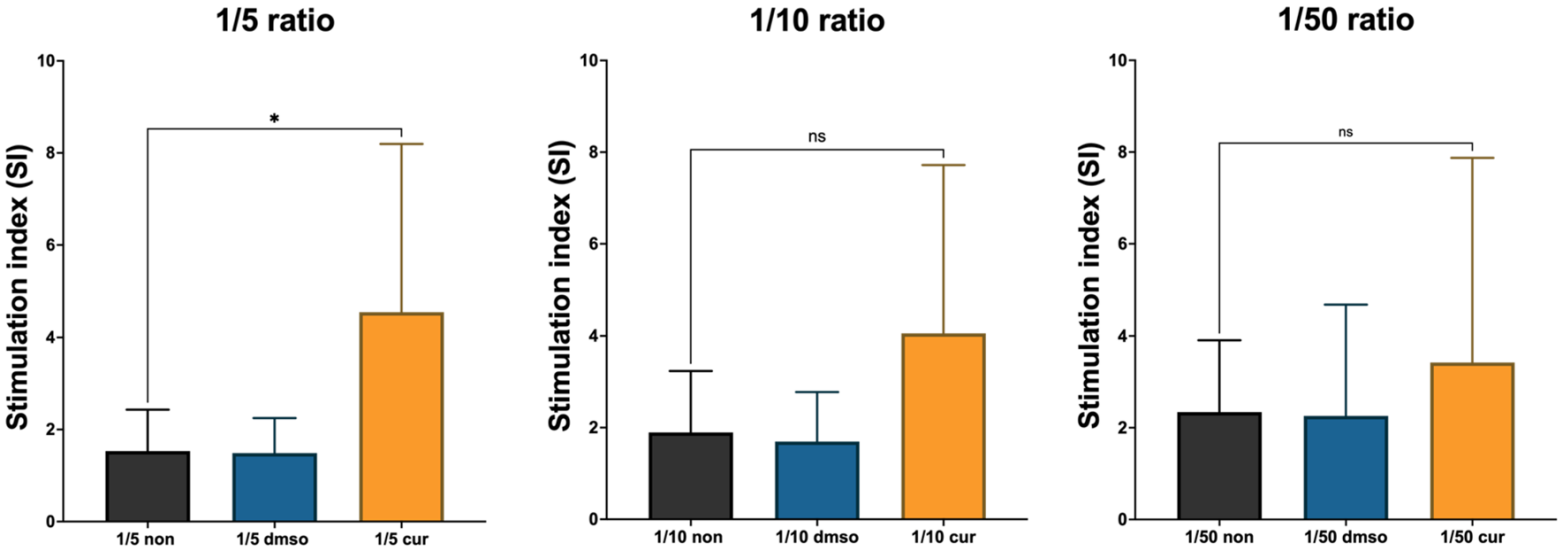
Increased Stimulation Index (SI) of PBMCs co-cultured with CAFs treated with Curcumin. The stimulation index of PBMCs co-cultured with corresponding CAFs and their respective control groups indicated that treatment with Curcumin restored the proliferation of PBMCs. However, this increase was not statistically significant. The highest SI was observed in the 1:5 ratio of CAF: PBMC co-cultures. As shown, in the no-treatment group, the highest SI corresponds to the lowest ratio, indicating the suppressive effects of CAFs. Data represents mean ± SD. Significant changes are indicated with asterisk *P-value < 0.05,

The changes in CAF’s function and its effects on PBMC responses were assessed using PBMC proliferation, cytokine production, and gene expression. Curcumin-treated CAFs and the respective controls were inactivated with Mitomycin-C and co-cultured with PBMCs in 3 different ratios. PBMCs were stimulated with PHA, and proliferation was measured using MTT assay. Figure 5 shows that treatment with Curcumin could restore PBMC proliferation; however, it was not statistically significant (P-value > 0.05).

**Figure 6:**
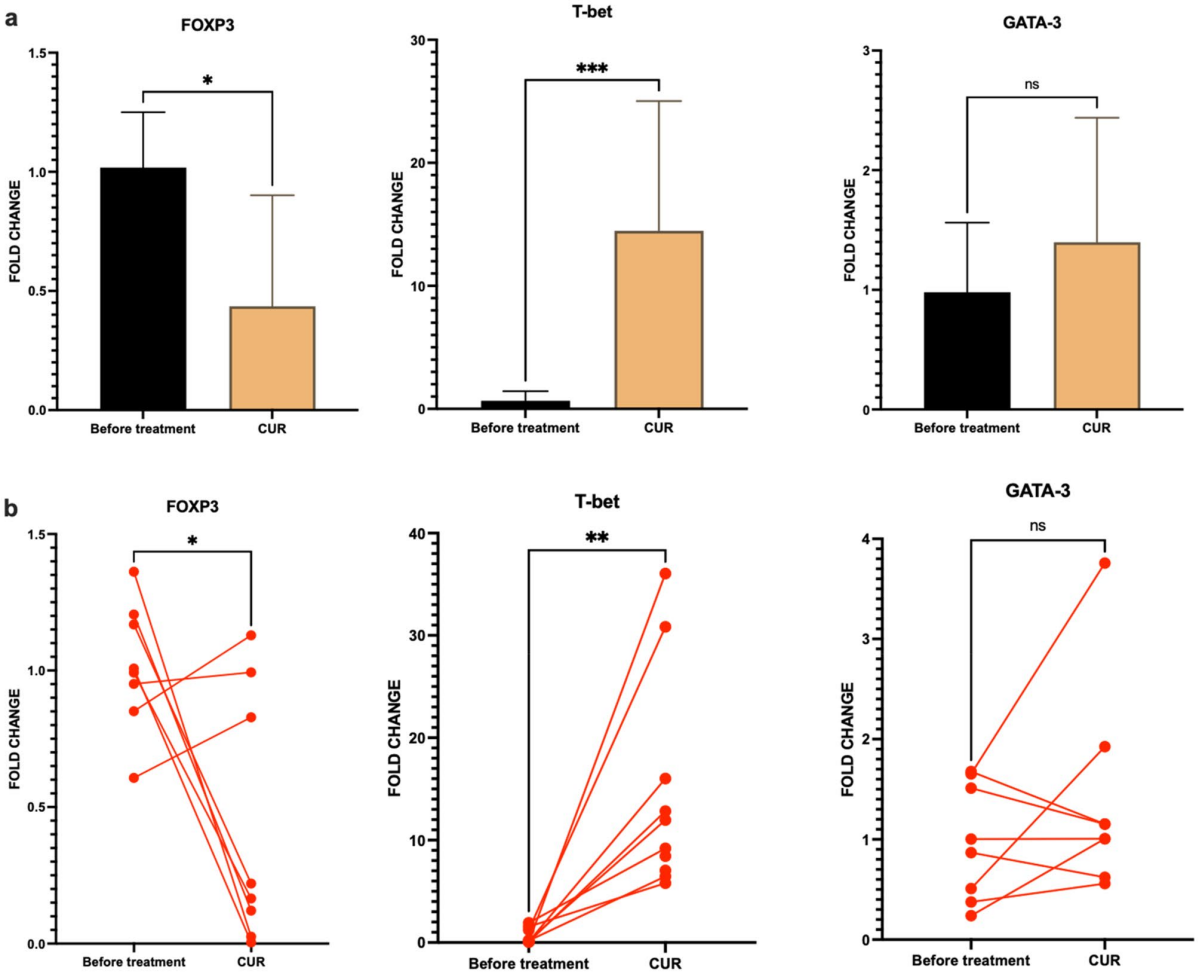
PBMCs gene expression analysis. The expression of transcription factors associated with Th differentiation, including FOXP3, T-BET, and GATA-3, was analyzed using Real-Time PCR. (**a**) Results indicated that treatment with Curcumin increased the expression of markers associated with Th1, including T-BET, and decreased the expression of markers associated with Tregs (FoxP3). (**b**) Individual changes in gene expression were analyzed with the paired-T test of 3 replicates. Results showed that treatment with Curcumin increased the expression of Tbet in all treated co-cultures efficiently. Data represents mean ± SD. Significant changes are indicated with asterisk *P-value < 0.05, **P-value < 0.01, ***P-value < 0.001.

**Figure 7:**
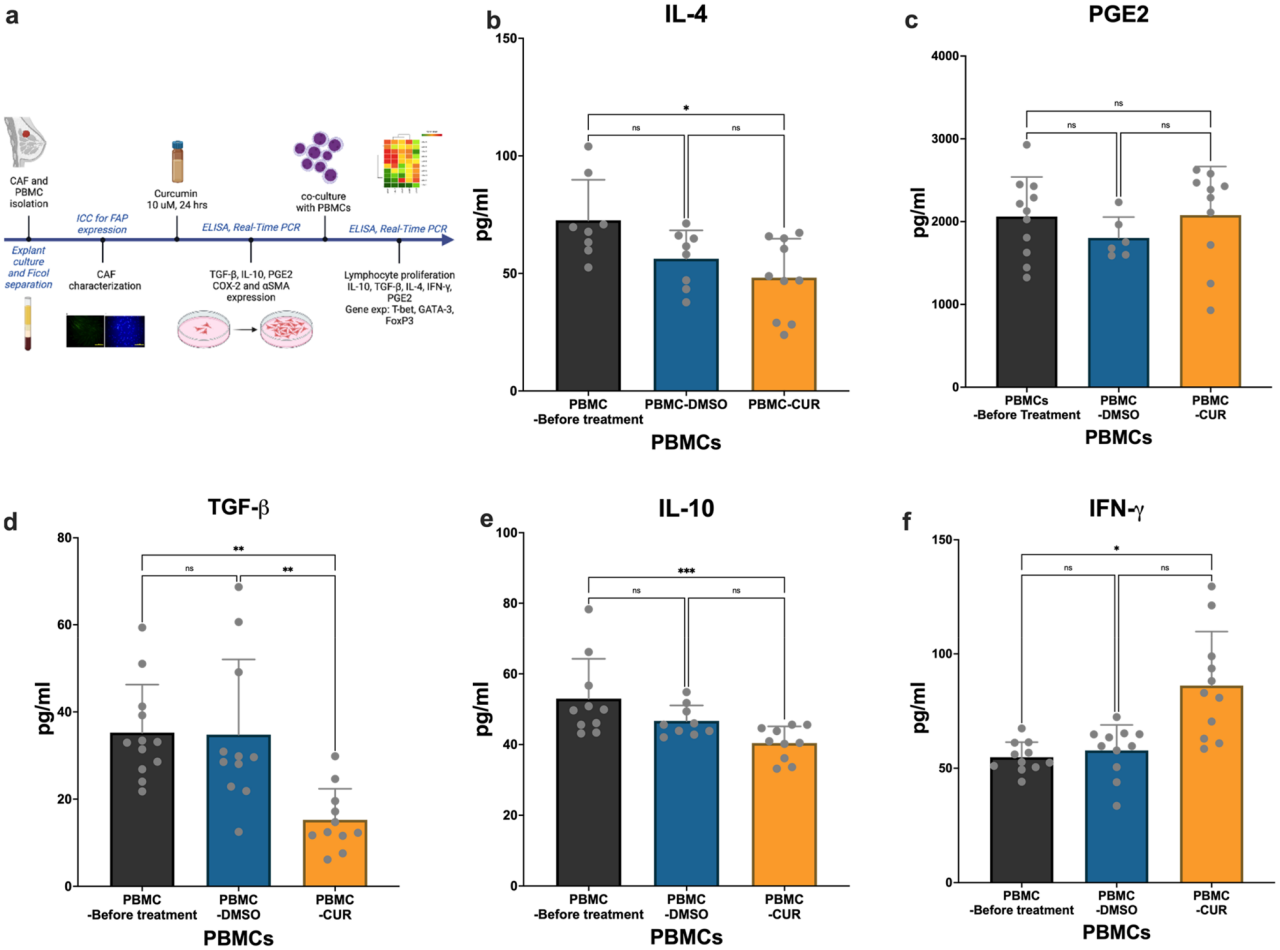
Cytokine production in co-cultures of PBMCs and Curcumin-treated CAFs. CAFs were isolated and treated with Curcumin. PBMCs were added, and the levels of cytokines were assessed in the supernatant after 48 h (**a**). The production of cytokines associated with Th differentiation, including IL-4, PGE2, TGF-β, IL-10, and IFN-γ (**b**–**f**), was evaluated in culture supernatants using ELISA. Results indicated that treatment with Curcumin increased the production of IFN-γ and reduced the production of TGF-βand IL-10, indicative of increased Th1 and inhibited Treg phenotype. Additionally, no significant change in PGE2 was observed. Data represent mean ± SD. Significant changes are indicated with asterisk *P-value < 0.05, **P-value < 0.01, ***P-value < 0.001.

The gene expression and cytokine profile of PBMCs were analyzed after co-culture with Curcumin-treated CAFs. Real-time PCR (Fig. 6) revealed that the mRNA of transcription factor T-bet increased 15-fold in PBMCs cultured with Curcumin-treated CAFs compared with the no-treatment controls (P-value = 0.004). In addition, the levels of FoxP3 mRNA decreased significantly in PBMCs (P-value = 0.03). The production of IFN-ɣ also increased in the supernatant of PBMCs co-cultured with Curcumin-treated CAFs (Fig. 7) (P-value = 0.01). This was followed by a decrease in TGF-β, IL-10, and IL-4 production (P-value = 0.001, 0.05, and 0.01, respectively). No alteration was observed in the expression of GATA-3 and PGE2 between the treatment and control groups (P-value = 0.7 and 0.5, respectively).

## Discussion

The dysregulation of protective inflammation leading to destructive inflammation is one of the culprits of increased incidence of inflammation-related diseases such as cancer^36^. Clinical and experimental studies indicate that chronic re-activation of inflammatory pathways leads to increased growth factor production, neo-angiogenesis, and immune suppression in the TME. Chronic or destructive inflammation management has shown protective effects in cancers such as colon^37,38^. However, in other types of malignancy, where the role of chronic inflammation is much less prominent, activation of inflammatory pathways can contribute to the onset and progression of cancer. Evidence has shown that destructive inflammation includes activating various signalling pathways, such as NF-kB, and the expression of inflammatory enzymes and cytokines^39^. COX-2, IDO, and iNOS are among the enzymes whose expression correlates with cancer progression^40–42^.

In addition to cancer cells, the cells of TME, including activated fibroblasts, contribute to the inflammatory milieu and immune suppression. CAFs are the principal stromal cells in the TME. It has been shown that compared to quiescent fibroblasts, CAFs express α-SMA and produce pro-inflammatory cytokines and COX-2, assisting tumor progression. Studies employing sorted flow cytometry and single-cell RNA sequencing have revealed subpopulations within the CAF population. With varying marker expression and production of crucial mediators, CAFs’ subpopulations are mainly the myofibroblastic myCAFs activated with TGF-β and the inflammatory iCAFs induced in NF-kB activation. myCAF and iCAF states are essentially unique. However, the two subpopulations are presumably to be interconvertible in response to TME, resulting in different cells in various activated polarizations, allowing an intermediate property with plasticity in CAFs^43^. Other subpopulations of CAFs have been described, including antigen-presenting MHC-II positive apCAFs, which also have an immunosuppressive property. In this study, a crude population of isolated CAFs was used. Although they may have phenotype diversities, CAFs show a common immunosuppressive property, which was the aim of Curcumin treatment, showing effects on both NF-kB activation and the TGF-β signaling pathway^44–47^.

Studies have shown that inhibition of COX-2 has direct anti-growth properties, and there is a close association between PGE2 and immune suppression. In colon cancer, PGE2 can suppress the function of various immune cells such as macrophages, neutrophils, Th1, CTL, and NK cells, and on the other hand, augment the activity of cells such as TSLP-dependent Th2, Th17, and Treg^48^. CAFs and their subtypes are also the primary producers of TGF-β in the cancer microenvironment^49,50^, which inhibits anticancer immune responses. Over-expression of immune-regulating cytokines such as TGF-β and VEGF by myCAFs induces Tregs in the stroma and creates a micro-inhibitory environment in Pulmonary adenocarcinoma with poor prognosis^15^. CAFs have been the target of therapy since their role as advocates of cancer growth has been established. Targeting the surface expression of FAP in a murine colon carcinoma model showed increased chemotherapy responsiveness and survival^51^. However, the results of studies targeting CAFs are inconsistent, and CAFs may have immune-beneficial properties depending on the cancer type^52^. Therefore, although the elimination of CAFs may not be feasible, however their function and mediators may.

The anticancer effects of Curcumin have been studied in a variety of cancers. Curcumin inhibits cancer-associated fibroblast-driven prostate cancer invasion, EMT, ROS production, and decreased CXCR4 and IL-6 receptor expression through MAOA/mTOR/HIF-1α signaling^53^. Curcumin increases the expression of p16 and inactivates the JAK2/STAT3 pathway, resulting in decreased expression of α-SMA and, consequently, the migration/invasion^54^, and suppresses NF-κB, Cyclooxygenase-2, and IL-8 expression in pancreatic carcinoma both in vitro and in vivo^55^. Curcumin, alone or in combination with Celecoxib, inhibited the growth of colorectal cancer cells in vitro by reducing COX-2 and PGE2^48^. Co-delivery of Curcumin and Doxorubicin in nano-micelles for Human non-small cell lung cancer, A549, significantly reduced NF-κB and COX-2 activity and tumor growth in lung carcinoma tumor-bearing mice while reducing the side effects of Doxorubicin^56^. In the present study, the expression of α-SMA and COX-2 genes in CAFs was reduced as a result of treatment with Curcumin. Consequently, the amount of PGE2 has been reduced in CAFs’ supernatant. However, Curcumin is not able to inhibit the COX-1 isoform, which is also expressed in CAFs^57^. Curcumin also shows immune-modulatory properties. In this regard, Shafabakhsh et al. observed that by inhibiting regulatory T cells (FoxP3 +) and shifting their phenotype to T helper 1 (T-bet +) cells, Curcumin was able to inhibit tumor growth^58^. Also, oral administration of Curcumin for one month in patients with advanced colon cancer significantly reduced FoxP3 expressing Tregs and increased T-bet expressing Th1 in the peripheral blood.^59^. In this study, it was observed that treatment with Curcumin reduced the production of TGF-β in CAFs. This decrease in TGF-β production may aid in reducing the suppressive microenvironment and the differentiation towards regulatory T cells and augment anti-tumor responses. Our study also showed that Curcumin treatment was also capable of reducing the expression of the FoxP3 in PBMCs. In addition, IL-10 was also reduced in PBMCs, which, together with a reduction in FoxP3 and TGF-β in PBMCs, could indicate reduced Treg function. On the other hand, we observed that the production of IL-10 in CAFs, another suppressive cytokine in TME, was unaffected by Curcumin. It should be noted that CAFs are not considered IL-10-producing cells in the tumor microenvironment^60,61^.

Qing Wang et al. observed that Curcumin at high doses (30, 40, 50 μM) has cytotoxic effects, but at low doses (1, 5, 10 μM) with a treatment duration of 48–72 h significantly increases lymphocyte proliferation^31^. In general, the PBMCs of cancer patients have lower proliferation potential and are in a suppressive state^62^. It is expected that alterations in dose and duration of treatment may benefit the alleviation of the immunosuppression of the tumor environment, which requires further investigation. Considering our results, it can be concluded that inhibition of PGE2 in CAFs increases the expression of the T-bet gene and the production of IFN-γ. Additionally, the suppressive microenvironment is alleviated by reducing the levels of the FoxP3 gene and reducing the production of cytokines IL-10, TGF-β, and IL-4. Decreased TGF-β and FoxP3 production in PBMCs may indicate decreased regulatory T phenotype. Therefore, by reducing inflammation in the tumor microenvironment, the pro-tumor phenotype is inhibited, and the anti-tumor phenotype is augmented in immune cells. Considering the results of this study, the limitations of this study was the missing in vivo analysis which can further our understanding of the anticancer mechanisms of curcumin.

Although Curcumin has numerous anticancer properties, its hydrophobic nature, biotransformation, and degradation limit delivery, bioavailability, and therapeutic effects^27^. Therefore, modulations in molecular structure or delivery systems have attracted much attention. Utilizing various nano-delivery systems such as dendrosomes^63^, nano-albumin^64,65^,  nano-chitosan^66^, nano-membranes^67^, and liposomes^68^ have shown increased Curcumin efficacy in experimental settings^69^. Also, the addition of a variety of decorations, such as folate receptor^70^, polyethylene Glycol^71^, and PD-L1^64^, can enhance targeted delivery to tumor tissue. On a broader perspective, the anti-inflammatory effects of Curcumin and Curcumin-derived formulations can have additional therapeutic indices in other inflammatory diseases where activated fibroblasts show immune-modulating effects such as fibrotic and autoimmune diseases such as rheumatoid arthritis and systemic sclerosis^72^.

## Conclusion

Considering the pivotal role of CAFs in the tumor microenvironment, one of the effective ways to treat cancers is to identify and inhibit tumor-promoting pathways in the TME^73,74^. The immune system plays an essential role in all types of cancer. Cancer cells evolve to escape the immune system. Regulatory T cells are a crucial component in tumor immune tolerance and are involved in the immune-escaping of cancer cells.

Even though many drugs have been approved for treating various types of cancer, none are entirely effective or free from side effects, especially for long-term applications. Curcumin can be very effective and free of severe toxicity^75^. Curcumin has multiple medicinal properties and can interact with several molecular targets and intracellular signaling pathways^76^. However, poor bioavailability limits its therapeutic effects. Curcumin analogs and their various formulations, including nanoparticles and liposomes, are extensively studied for maximum effectiveness.^77,78^. For successful cancer treatment, well-controlled and complete clinical trials must be performed in more significant numbers to bring this very effective and promising factor to the forefront of cancer treatment.

## Data Availability

The data supporting this study's findings are available from the corresponding author upon request.

## Abbreviations

α-SMA: Smooth muscle actin-alpha
BCL-2: B-cell lymphoma 2
BCL-XL: B-cell lymphoma-extra large
CAFs: Cancer-associated Fibroblasts
COX-2: Cyclooxygenase-2
DMSO: Dimethylsulfoxide
FAP: Fibroblast Activation Protein
FoxP3: Forkhead box-p3
GAPDH: Glyceraldehyde 3-phosphate dehydrogenase
GATA-3: GATA Binding Protein 3
HIF-1α: Hypoxia-inducible factor 1-alpha,
IDC: Invasive ductal carcinoma
IFN-γ: Interferon gamma
IL-10: Interleukin-10
IL-4: Interleukin-4
INOS: Inducible nitric oxide synthase
MMPs: Matrix metalloproteases
MTT: [3-(4,5-Dimethylthiazol-2-yl)-2,5-diphenyltetrazolium bromide]
NF-Κb: Nuclear factor kappa-light-chain-enhancer of activated B cells
NSAIDs: Non-steroidal anti-inflammatory drugs
PBS: Phosphate-buffered saline
PBMCs: Peripheral blood mononuclear cells
PGE2: Prostaglandin E2
PHA: Phytohaemagglutinin
RPMI: Roswell Park Memorial Institute Medium
STAT3: Signal transducer and activator of transcription 3
T-bet: T-box protein expressed in T cells
TGF-β: Transforming growth factor-beta
TME: Tumor microenvironment
Treg: Regulatory T cells
VEGF: Vascular endothelial growth factor
